# Tracking free water in human body by magnetic resonance imaging: new insights on the network pathways

**DOI:** 10.1098/rsos.250065

**Published:** 2025-06-11

**Authors:** Lu Sun, Liting Wang, Le He, Changsong Liu, Fengshan Bai

**Affiliations:** ^1^ Department of Mathematical Sciences, Tsinghua University, Beijing 100084, People’s Republic of China; ^2^ Department of Electronic Engineering, Tsinghua University, Beijing 100084, People’s Republic of China; ^3^ School of Medicine, Tsinghua University, Beijing 100084, People’s Republic of China

**Keywords:** free water, network pathway, non-invasive, physiological mechanism, interstitial space

## Abstract

Water is the most indispensable material for life. Although extensive research has been carried out at the microscopic level, including studies focusing on transport and molecular effects, the macroscopic water system of the human body is still unclear. In this study, the distribution of free water in the human body under natural conditions was non-invasively depicted by magnetic resonance hydrography sequences. Spatial saturation technique was used to explore the dynamic properties of free water. Imaging and post-processing results reveal that abundant free water formed a macroscopic network consisting of interconnected pathways. The slow flow trajectories of some waterways were captured in the saturation band. The free-water channels enclosed or bordered the space around blood vessels and nerves or traversed the tissues. This spatial relationship is similar to the fluid flow patterns in the interstitial space in the periphery and the glymphatic system of the brain, suggesting potential physiological functions and pathological variations.

## 1. Introduction

From the propagation of species on Earth to the exploration of living organisms in outer space [1], water is central to life, sustaining cellular life across different scales of time and distance [2]. It is the most abundant substance in the human body, forming 50–80% of the body [3], and can be divided into free water and bound water according to its motion ability. The statistical relationship between free and bound water in specific tissues [4], especially under pathological conditions [5,6], has aroused research interest. However, little attention has been paid to the accumulation and location of free water, which cannot be characterized by proportional values. At the macroscopic level, the characteristics of free water in the human body are still not well understood.

The peripheral tissues are infiltrated by fluid, especially abundant interstitial fluid. A fluid-filled space was found in the submucosa of human tissues, within and between organs, and in the perineurium and vascular adventitia [7,8]. This space is connected to lymph nodes [5] supported by a complex network of collagen bundles. Other studies have also confirmed that the interstitial space is continuous and filled with flowing fluids. Li *et al*. observed tracer fluid pathways around the veins of healthy rabbits [9] and in the connective tissue of lower limb amputees [10] by magnetic resonance imaging (MRI) and fluorescein photography. Han *et al*. injected liquid metal [11] and gold nanoparticles [12] into the intrathecal space of rats, with the former wrapped around fascia fibres and the latter delivered to the brain through the perivascular space of the carotid artery and blood vessels. Benias *et al*. [7] also described the fluid-filled space as a pre-lymphatic space, a concept that had been proposed earlier. This space is likely involved in liquid transport [13]. However, most studies were carried out at the microscopic level, and the fluid-filled space in the periphery has not been well demonstrated at the macroscopic level.

There are several brain-wide fluid transport pathways in the brain [14,15], known as the glymphatic system. It has functional and structural similarities with the peripheral interstitial space. Periarterial [16,17], perivenous [14] and perineural spaces [18,19], as well as meningeal lymphatic vessels [20], are widely discussed as possible transport compartments. Extensive research on the glymphatic system can provide a technical model for the exploration of peripheral interstitial space.

MRI can effectively explore the static and dynamic properties [15,21,22] of free water in the human body. However, the MRI findings are influenced by changes in anaesthesia and invasive state [6,23], differences in animal anatomy [6] and pressure and volume sensitivity of tracer [24]. Taking these factors into consideration, we performed three-dimensional (3D) GRASE MRCP sequence (gradient and spin-echo magnetic resonance cholangiopancreatography) [25,26], an MR hydrography technique, to provide high-resolution images of the distribution of free water in the human body in the natural state. Saturation bands were introduced to obtain dynamic information about the slow flow.

**Figure 1.**
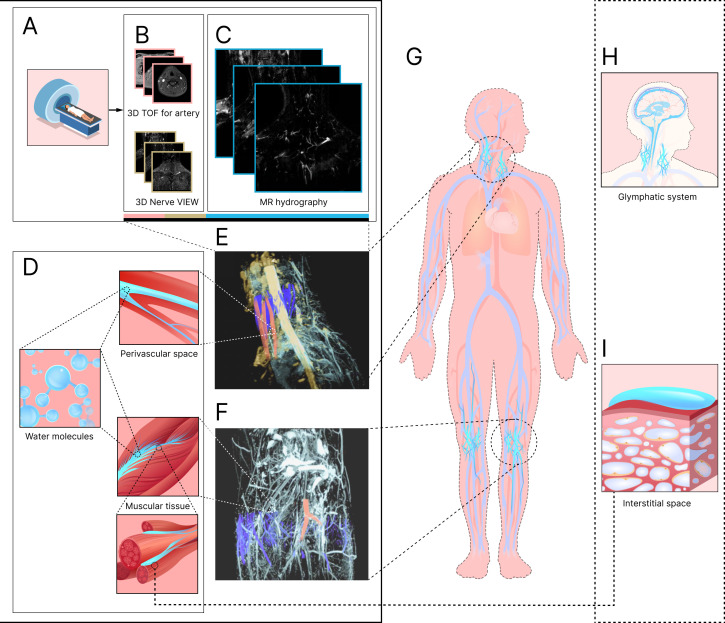
Macroscopic free-water network of human body visualized by MRI. (A) Schematic and main imaging sequences of MRI of the neck: 3D TOF (time of flight) and 3D Nerve VIEW depicted blood vessels and nerves (B), and MR hydrography characterized free water (C). (D) Multiscale analysis of water. Microscopic water molecules flowed in the local blood vessels or local interstitial spaces and constituted a macroscopic network. The free-water network in the nape (E) and knee (F), as well as arteries, veins and nerves, was depicted by 3D MR reconstruction. These regions were interrelated in spatial structure and distribution. (G) Locations of the observed water network in the human body. Previously studied glymphatic system in the brain (H) and fluid-filled interstitial space (I) in the periphery. Light blue, free water; dark blue, vein; red, artery; yellow, nerve.

In this study, we depicted the macroscopic free-water network in the human body, considering the nape and lower leg, including the knee, as the representative imaging areas in figure 1. Modified 3D GRASE MRCP sequences (MR free water (MRF)) were acquired to explore the structure and morphology of free-water pathways in healthy subjects under natural conditions. The feasibility of using the space saturation technique to characterize the flow information of free water with postulated slow movement was also studied. In addition, arteries, veins, nerves and tissues were depicted using regular sequences, and the spatial distribution of water channels within these tissues was visualized through image reconstruction and fusion by 3D Slicer software [27] and synchronous anchor points of two-dimensional (2D) images. The results showed that the free water formed a large-scale network, consisting of interlaced and interconnected pathways. Fluid in the waterways displayed fluidity in the saturation band. The spatial distribution of the network was highly matched with arteries, veins, nerves and tissues. Similar structural and morphological associations exist in the interstitial space and the glymphatic system, suggesting potential functional consistency and indicating the free water pathway as part of the pre-lymphatic channel.

## 2. Results

### 2.1. Re-emergence of free water in network mode

**Figure 2.**
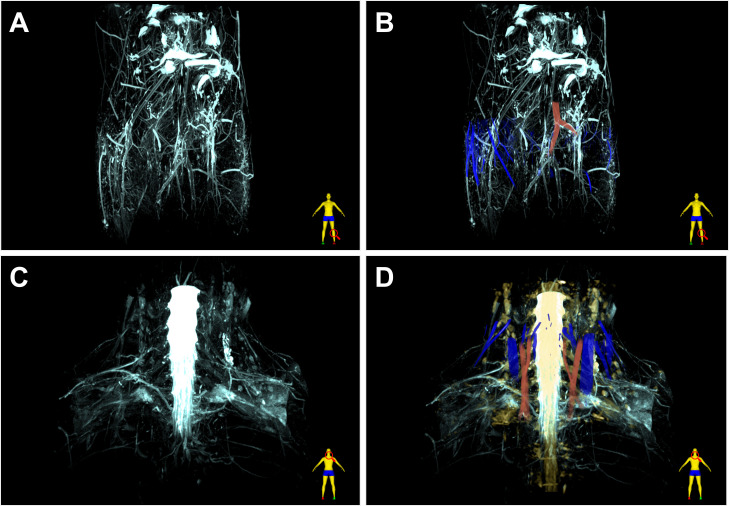
Fusion 3D images of free water pathways, arteries, veins, nerves and tissues at different sites. (A,B) Lower leg with knee. (C,D) Nape. Light blue, free water; dark blue, vein; red, artery; yellow, nerve.

The free water visualized by the MRF sequence displayed specific distribution patterns and characteristics, as shown in 3D reconstructed images and videos (figure 2A,C; electronic supplementary material, videos S2 and S4). It was part of the networks observed in the human body, as illustrated in the area of the lower leg and nape. The free water at the scanning site radiated outward from the centre of the field of view (FOV) or congregated inward from the outer surface. The signal intensity was highest in the middle of the field, corresponding to the knee synovial fluid or the spinal fluid, respectively. At the edge of the network, the free water emitted a weaker signal, whereas the branching of the water network increased, suggesting a tendency toward a looser structure near the body surface.

The macroscopic network structure was subdivided into interconnected and ordered free-water channels. These pathways typically appeared smooth and continuous, with some exhibiting the characteristics of parallel tubes. A part of the channels was confirmed to be perivascular or perineural pathways by the fusion of 3D image reconstruction. Other parts exhibited a more circular or swirling appearance, unlike a straight shape. Near the network centre, the water channels were characterized by a wider and smoother trajectory. Furthermore, the pathways became narrower and had various shapes closer to the body surface. Moreover, a small amount of free water was visible at scattered points, which were found mainly near the bottom layer of the network.

### 2.2. Visualization of spatial distribution relationships between free water pathways and other structures

**Figure 3.**
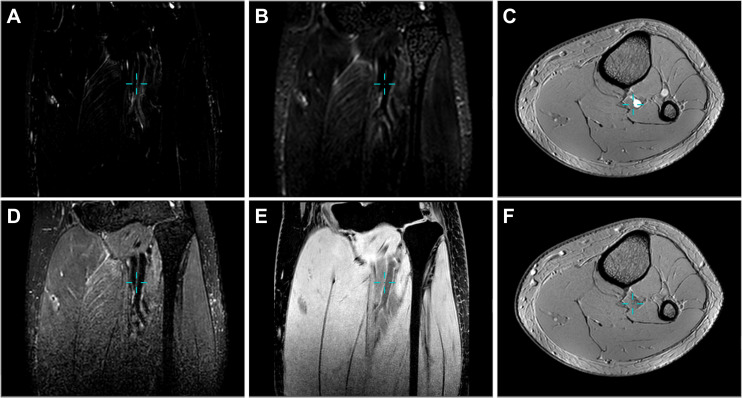
2D slice images of the lower leg, including the knee, from different 3D sequences, with the synchronized anchors (blue crosses) pointing towards the edge of the tibial nerve and the posterior tibial artery. (A) MRF (MR free water). (B) 3D Nerve VIEW. (C) 3D TOF (time of flight) for arterial angiography. (D) VISTA (volume isotropic turbo spin echo acquisition). (E) PROSET (principle of selective excitation technique). (F) 3D TOF for venous angiography.

The familiar, classical and widely distributed tubular structures in the human body, including the vasculature and nervous system, were delineated for morphological analysis of the free water network. Fusion images (figure 2) of free water pathways and these structures were reconstructed using 3D Slicer software to visualize the spatial connections. The corresponding 3D views are presented in electronic supplementary material, videos S2–S5. Considering that the readability would be reduced if the reconstructed image also incorporates tissue components, we described the distribution relationship between free water and tissue by 2D section and synchronous anchor points (figure 3E; electronic supplementary material, figures S1E, S2E and S3E). For the reconstruction of the tibial nerve in the leg, we depicted it in a 2D profile for similar reasons. However, in the nape, a more comprehensive neural image was obtained using the 3D Nerve VIEW sequence and incorporated into the reconstruction, as shown in figure 2D.

The perivascular space is an important destination of free water, as demonstrated by 3D fusion reconstruction near the lower leg. In particular, in figure 2B, several tube-like water channels (light blue) near the body surface precisely overlapped or bordered veins (dark blue). The posterior tibial artery and the downstream fibular artery were also surrounded by free water. In electronic supplementary material, figure S1, the crosses point toward the venous edge, as confirmed by venous identification at the transverse plane of 3D TOF and coronal vascular wall imaging of volume isotropic turbo spin echo acquisition (VISTA) sequence, whereas the MRF image depicts a section of the perivenous passage composed of free water. Similarly, in figure 3, the location markers indicate arteries, as evidenced by 3D TOF (figure 3C), VISTA (figure 3D) and principle of selective excitation technique (PROSET) (figure 3E) imaging, whereas the filamentous waterways appeared in the corresponding sites in the MRF image (figure 3A).

The perinervous space was also a preferred site for free water. The PROSET sequence described the position of the anchor point in figure 3E, located between the tibial nerve and the posterior tibial artery. Furthermore, the cross in electronic supplementary material, figure S2e, is placed in the region beside the tibial nerve, away from the artery. The tube-like traces of free water were observed in both places with the signal more pronounced in the latter space. The free water pathways of the posterior neck were highly overlapping and connected to the cervical plexus, as shown in figure 2D and electronic supplementary material, video S5.

Free water pathways also appeared in interstitial spaces, such as perineural spaces. Electronic supplementary material, figure S3, shows another typical case of free water distribution in tissues. The 3D time of flight (TOF; electronic supplementary material, figure S3c,f) sequence ruled out the possibility that the anchor point was located near blood vessels. Furthermore, PROSET demonstrated that the anchor point was located in the interstitium of muscle tissue (electronic supplementary material, figure S3e). In particular, in the MRF sequence shown in electronic supplementary material, figure S3a, in addition to the significant continuous channel between the muscle groups, feathered waterways extending along the main route were visible, indicating a regular pattern of free water distribution in the muscle fibres.

### 2.3. Free water depicted by magnetic resonance free water

**Figure 4.**
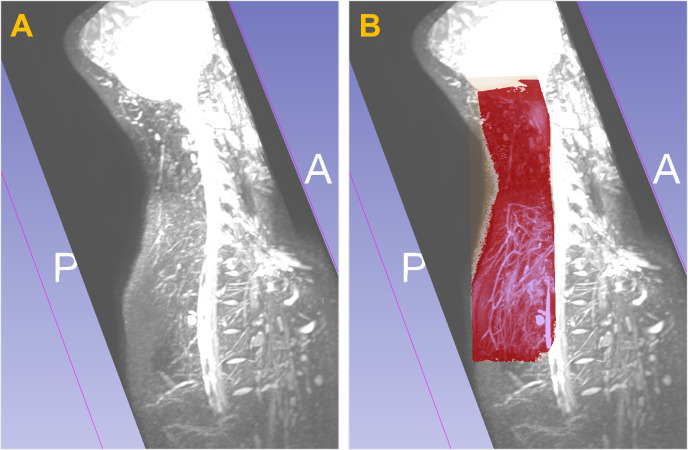
MIP (maximum intensity projection) image in the sagittal plane of the nape using 3D Nerve VIEW sequence (A) and superimposed MRF (MR free water) sequence (B, red).

MRF exhibited a remarkable ability to fully characterize the free water network structure and channel morphology. Figure 3 and electronic supplementary material, figures S1–S3, depict the MRF (figure 3A), 3D Nerve VIEW (figure 3B) and VISTA (figure 3D) images. These images were obtained from three sequences with long echo (TE) times, suggesting different levels of water imaging effect. MRF technique specializes in free water imaging, which produces the most concise images with extremely high contrast. The VISTA sequence can characterize the contours of vascular walls, utilizing the flow void effect. The 3D Nerve VIEW sequence targets the cervical or brachial plexus (figures 4 and 2D) but has limited ability to depict the nerves in the legs. Compared with MRF imaging, the VISTA and Nerve VIEW sequences reproduced only part of the free water, overlapping several components not being evaluated in this study.

Compared with a free water image, signal in the saturated area in electronic supplementary material, video S1, was almost suppressed. However, some filamentous traces explicitly remained in the black band. Notably, these findings indicate the fluidity of the liquid in the 2D slice. The flow direction is determined by the change in the observed fluid in the image combined with the front and back sections. The filamentous fluid pathways that partially penetrated the saturated areas typically extend from the foot to the head, consistent with the direction of venous flow.

The sagittal MIP image for the neck in the 3D Nerve VIEW and superimposed MRF sequences are shown in figure 4. This imaging comparison provides comprehensive information regarding free water distribution similar to MRF (figure 4B, red).

## 3. Discussion

In this study, we modified MRF to visualize free water in the human body. The widely distributed free water formed a network of pathways, which surrounded, bordered or travelled through arteries, veins, nerves and tissues. The flow characteristics of free water were preliminarily demonstrated using the saturation technique. The continuous structure and spatial distribution provide new evidence on the continuity of interstitial space [7,8](electronic supplementary material, S2 and S3). Earlier anatomical and tracer imaging experiments have demonstrated the pervasiveness of fluid-filled interstitial spaces [7–12,28]. For example, Benias [7] and Cenaj *et al*. [8] demonstrated the structure and continuity of free water across tissue and organ boundaries, including fascia, perivascular soft tissue and perineurium.

The direct connection of free water channels with vasculature, nerves and tissues indicates the role of the ‘pre-lymphatic’ channels [7,8,13]. The existence of pre-lymphatic channels is controversial [13], referred to as ‘low-resistance pathways’ in skin and muscles that are responsible for fluid transport from capillaries to initial lymphatic vessels. At the microscopic level, the continuity of the interstitium and lymphatics has been directly or indirectly confirmed in human tissue sections and animal anatomical experiments [5,7,8]. The pre-lymphatic channels of the breast have been observed with direct anastomoses of the true lymphatics [29], and even possible pathways for tumour spread [30]. However, from the macroscopic perspective, evidence of the existence of pre-lymphatics in the human body under natural conditions is insufficient. The free-water network pathways may fill the gap. As there are no typical beaded [31] or honeycomb [32] features, and there is no lymphatic disease, it can be assumed that the peripherally visible water is not lymphatic fluid. Nevertheless, given the fact that lymphatic vessels are usually not visible on MRI in healthy subjects due to the very narrow ducts and the abnormal changes in the fluid routes under pathological conditions [5,6], it is challenging to delineate both the free water pathways and the lymphatic channels in the natural state without administering contrast agents. Abnormal changes in the visualized pathways may be directly related to pathological states, such as inflammation [4,5], secondary peripheral oedema, tumour [5], lymphatic failure [6] and ligament damage [33]. However, unlike the relatively stable water homeostasis in the brain, free water in the peripheral interstitial space has greater variability. Therefore, age [4] and short-term factors, such as sleep and exercise, could affect the network pathways more rapidly and to a larger extent.

The spatial relationship between the water channels and other structures suggests a formation basis. The active movements of free water make it challenging to independently form such a large-scale, well-ordered network pattern. Chambers formed by stable connective tissues surrounding vessels and nerves [9,10,14,17–19,21], including various membrane structures, as well as tissue spaces [7,8], are ideal sites for free water flow and aggregation. This spatial regularity is also embedded in the glymphatic system of the brain [14,16–20] providing a theoretical basis for the formation mechanism. Classical driving forces [8], including pulsation effects, pressure differences and breathing movements, can be derived from various hypotheses related to the glymphatic driving model in the brain [34–36]. However, precise models accounting for the formation of free water networks require more detailed data and experiments in the future.

The improved hydrography method (MRF) accurately depicted the abundant free water in the human body. The MRF, VISTA and 3D Nerve VIEW sequences can depict water in principle (figure 3 and electronic supplementary material, figures S1–S3) but the latter two incorporate specialized modifications. With the technical improvements and imaging requirements, VISTA and 3D Nerve VIEW images discard secondary information about free water. In addition, the 3D spatial MR imaging method is superior to histological analysis in terms of imaging range, time benefit, repeatability and fluid route identification, both for the brain [35] and the periphery. Lee *et al*. [23] demonstrated that waking or anaesthetic states, as well as postures during sleep, may affect the efficiency of glymphatic transport. In traditional tracer imaging experiments, the normal flow direction may be altered by the pressure caused by injecting the tracer into the brain [24]. Furthermore, the results may vary with the molecular sizes of the agent. The methods and results of animal experiments in rats and rabbits may not be generalized to humans, and the fluid distribution in the interstitial space of subjects under anaesthesia or in inflammatory states such as peripheral oedema [6] may exhibit artifactual or pathological changes. Moreover, the traditional MRCP technique produces static water images. To reveal the dynamic characteristics, we introduced the saturation band as the background plate. High-speed movements such as those of blood are not visible due to the flow void effect, and flow at appropriate speeds through saturated areas can be identified (electronic supplementary material, video S1).

**Figure 5.**
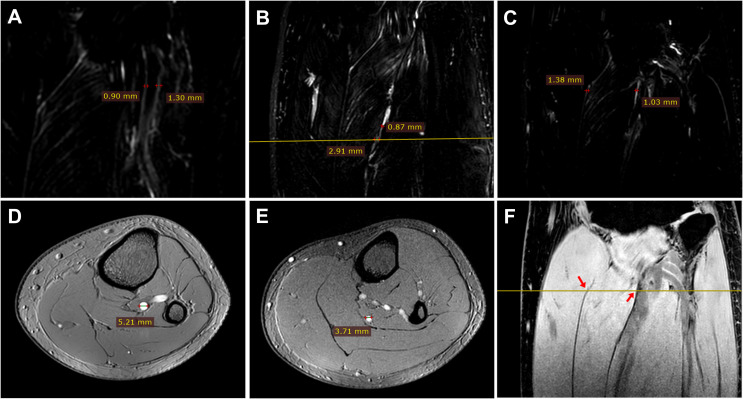
Markers of the size of typical water channels and vessels from different 3D sequences at the lower leg region including the knee. (A–C) MRF (MR free water). (D) 3D TOF (time of flight) for arterial angiography. (E) 3D TOF for venous angiography. (F) PROSET (principle of selective excitation technique). The diameter of an artery is marked in the transverse plane in (D), with the surrounding free water pathways marked in the coronal plane in (A). The width of a vein is marked in transverse plane in (E), with adjacent (yellow line) and upstream free water channels marked on the coronal plane in (B). The water channels between the musculature on the yellow line in (F) are marked in (C) on the same coronal plane, indicated by red arrows.

A major drawback in this study is related to the resolution and varying sizes of free water channels. Three-dimensional MRCP [26] and heavily T2-weighted [37] sequences enable water imaging at the millimetre and sub-millimetre levels. Considering the acquisition time and other factors, the voxel size in the MRF sequence of this study was set at $0.65\times 0.65\times 1.2\;mm^{3}$. Figure 5 marks the width of water channels and blood vessels. The marked free water channels surrounding the vessels and inside the muscle tissues had widths in millimetres. However, the pathways vary widely in size, shape and location, often with unclear boundaries. It is challenging to accurately quantify the size of these channels. Notably, the size of free water channels observed by MRF differs from that determined by microscopic experiments, which usually exhibit water layers at the microscopic scale [7,8]. Although the presented imaging scheme does not achieve accuracy at the microscopic level by MRI, it can depict the macroscopic, complex water networks with lengths in centimetres and width in millimetres.

Several other limitations of the study should also be considered. The study did not perform adequate quantitative or statistical analysis. Differences between subjects and between various observation times in the same subject may provide additional information about free water accumulation and location. The avoidance of contrast agents increased the challenges of fluid quantification. Additionally, there were few subjects, with insufficient representation of various characteristics. Larger and more diverse population samples are required to obtain generalizable conclusions and facilitate standardized MRI scanning procedures. Furthermore, we anticipate to calculate the flow velocity from the distance of the liquid rushing into the saturation band and the excitation and scanning times. However, the calculation of the time parameter is still in the experimental stage. The preliminary flow information, such as direction, has only limited value. In the previous experiments, we implemented 2D phase-contrast sequences with quantitative flow function but the results were unsatisfactory due to the small size of the water channel and the presence of artefacts. Furthermore, lymphatic vessels are typically difficult to image without contrast agents. The relationship between lymphatic vessels and free water pathways in this study is a reasonable deduction based on previous similar structures and related conclusions. For both imaging techniques, strategies to minimize the influence of contrast media and physiological state require further consideration.

In addition to addressing the aforementioned limitations, future studies should include a more comprehensive exploration. Sequence parameters and 3D models should be further modified to determine the spatial relationship between free water channels and vasculature, nerves, tissues and possibly lymphatic channels. Subsequent experiments should explore continuity on time scales, i.e. dynamic relationships between different fluids, to explore water cycling. Moreover, we plan to extend the observation sites in this study to the complete free-water network throughout the body. The relationship with the traditional Chinese medicine meridian system should also be explored in future research. By analysing the variations in free-water pathways in ageing and pathological conditions, auxiliary diagnosis, disease analysis and new treatment strategies can be developed in the context of big data and modern health.

## 4. Conclusion

In summary, we identified the macroscopic free-water networks in the human body, focusing on the lower limb and nape region. The basic properties and morphological structures of water channels were verified by MR hydrography and 3D reconstruction techniques. In the observation area, the complex water network was found to be interwoven, connected and overlapping with arteries, veins, nerves and tissues, forming an extensive and orderly spatial structure. Slow flow was demonstrated in some water pathways. We speculate that this extensive network is closely related to the structure and function of the glymphatic system in the brain and the interstitial space in the periphery. Our findings enhance the understanding of the physiological mechanisms and pathological interpretation of living organisms.

## 5. Methods

### 5.1. Magnetic resonance imaging acquisition

This study was approved by the Tsinghua University Medical Ethics Committee. All participants provided written informed consent for the study (4 males, 10 females; mean age 26.2 years). None of the participants had any underlying conditions (such as heart disease), contraindications to MRI or conditions that might alter the results (such as lymphatic diseases and peripheral nervous injury).

Images were acquired on a 3T MR unit (Philips, Best, The Netherlands) using a 20-channel head–neck coil and an 8-channel knee coil. The superior segment of the lower leg, including the knee, as well as the nape, was the primary observation site. We performed a modified version of MRCP sequence, which evolved from heavily T2-weighted imaging techniques to strongly contrasted slow-moving liquids with faded background tissue, taking advantage of the long T2 relaxation time of the fluid [25]. Scanning acquisition mode of the thin-layer 3D imaging was applied to obtain images with a high signal-to-noise ratio at a spatial resolution of 1 mm in humans [26] or phantoms [38]. The representative 3D gradient and spin-echo (GRASE) technique can achieve higher MRCP image quality with a shorter acquisition time compared with the classic 3D turbo spin echo sequence [39,40]. The specific imaging parameters of MRF were as follows: repetition time (TR) = 1500 ms, TE = 160.4 ms, flip angle (FA) = $90^{\circ }$, FOV = $180\;\times \;180\;\times \;90\;mm^{3}$, voxel size = $0.65\;\times \;0.65\;\times \;1.2\;mm^{3}$ and slice thickness = 1.2 mm.

Additional MRI sequences were used to compare the imaging effects of several conventional hydrography-related techniques while characterizing the structures formed by free water pathways in relation to blood vessels, nerves and tissues. Both VISTA and 3D Nerve VIEW sequences include long echo times and therefore, in principle, can provide images of the water. In practice, these techniques have been refined to image target components, with the former more capable of delineating blood vessels and the latter imparting high signals to nerves. We performed these two sequences to emphasize the purity and integrity of the MRF image (VISTA: TR = 2000 ms, TE = 237.2 ms, FA = $90^{\circ }$, slice thickness = 0.8 mm; Nerve VIEW: TR = 2200 ms, TE = 170 ms, TI = 250 ms, FA = $90^{\circ }$, slice thickness = 2 mm).

On the other hand, specific sequences were selected to characterize the structure and distribution of free water channels in relation to blood vessels, nerves and tissues. Considering the advantages of the high resolution of 3D imaging and subsequent image reconstruction, 3D TOF sequences were performed to distinguish arteries and veins (TR = 30 ms, TE = 4.8 ms, FA = $15^{\circ }$, slice thickness = 2 mm). The cervical plexus was depicted with the conventional 3D Nerve VIEW sequence. Images of the popliteal nerve and tissues (mainly muscle) near the knees were simultaneously acquired by an additional PROSET sequence (principle of selective excitation technique; TR = 8.5 ms, TE = 4.6 ms, FA = $10^{\circ }$, slice thickness = 1.2 mm).

Spatial saturation technique was incorporated into the MRF sequence to determine the fluidity of free water pathways and identify possible flow states. For slow-moving fluids, quantifying the flow velocity in non-contrast-enhanced MRI is challenging. The high-speed flow in large vessels can be characterized by conventional phase contrast sequences, but the slow flow in narrow vessels is difficult to identify. In clinical settings, spatial saturation techniques are performed to eliminate signals from outside the areas of interest, or to eliminate artefacts, including those related to pulsations and breathing. However, we placed a saturation band in the FOV to suppress signals within it. The slow-flowing liquid outside the target region with an undiminished signal was visualized when it flowed into the black region of the saturation band. The orientation and distance to the saturated area provide information about the flow, including the direction and velocity. For instance, a single saturation band (thickness = 30 mm, power = 1) was placed perpendicular to the lower leg, as shown in electronic supplementary material, video S1.

### 5.2. Imaging processing and analysis

The maximum intensity projection (MIP) algorithm is one of the most widely used methods for image reconstruction, providing intuitive visualization of the trajectory and continuity of channels (such as blood vessels and nerves). After identifying the general structure in the reconstructed MIP images, we further explored the static spatial relationships between free water pathways and blood vessels, nerves and tissues using 3D Slicer software [27]. This software can fuse images of different 3D sequences simultaneously, restoring detailed information about spatial distribution. This allows for a more intuitive and effective interpretation of the spatial relationships between free water, blood vessels, nerves and tissues by MR sequences, including information regarding the location, distance and density.

The combination of the free water imaging technique and the saturation band provides an alternative strategy to evaluate fluid dynamics. During MRF scanning, the arteriovenous blood flow signal in the saturated area of the visual field is suppressed, and the slow movement of free water along the pathway may be identified. In particular, the orientation and distance of the fluid relative to the saturated region provide information about flow direction and speed. For example, if a trace of fluid is observed in the lower leg moving from the foot toward the saturation band, it can be assumed that the fluid flows in line with the direction of the vein. The flow velocity is positively correlated with the fluid length that enters the saturated region.

## Data Availability

The raw data are in DICOM format and contains personal information about the subjects. In order to protect the privacy of human subjects and comply with their wishes, we present part of the (2D) scan results in the form of video. One of this work's contributions is the establishment of standardized free water scanning processes, which can help researchers conduct validation and generalization. Sample data can be found at Dryad [41]. Supplementary material is available online [42].
